# Supplementary material for: Fabrication of Robust Capsules by Sequential Assembly of Polyelectrolytes onto Charged Liposomes
Source: Langmuir. 2021 May 4;37(20):6189–200. doi: 10.1021/acs.langmuir.1c00341 (PMC9205565; doi:10.1021/acs.langmuir.1c00341)
Supplement: Supplementary file 1 — la1c00341_si_001.pdf [file la1c00341_si_001.pdf]

Supporting Information for
Fabrication of robust capsules by sequential assembly of polyelectrolytes onto charged liposomes

Marta Ruano[1,†], Ana Mateos-Maroto,[1] Francisco Ortega[1,2*], Hernán Ritacco[3], José E. F. Rubio[4], Eduardo Guzmán[1,2], Ramon G. Rubio[1,2]

[1] Departamento de Química Física, Facultad de Ciencias Químicas, Universidad Complutense de Madrid, Ciudad Universitaria s/n, 28040-Madrid, Spain
[2] Instituto Pluridisciplinar, Universidad Complutense de Madrid, Paseo Juan XXIII 1, 28040-Madrid, Spain
[3] Instituto de Física del Sur (IFISUR)-Universidad Nacional del Sur, Av. Alem 1253, 8000-Bahía Blanca, Argentina.
[4] Centro de Espectroscopia y Correlación, Universidad Complutense de Madrid, Ciudad Universitaria s/n, 28040-Madrid, Spain

[†]Current address: Strathclyde Institute of Pharmacy and Biomedical Sciences, University of Strathclyde. 161, Cathedral Street, Glasgow, Scotland, UK G4 0XU

Corresponding Author: fortega@ucm.es

TABLE OF CONTENTS

Page

Figure S1: Amount of polyelectrolyte for overcompensation S2

Figure S2: Overcompensation ratio S3

Figure S3: DLS and zeta potential distributions S4

Figure S4: Set of ESR spectra S5

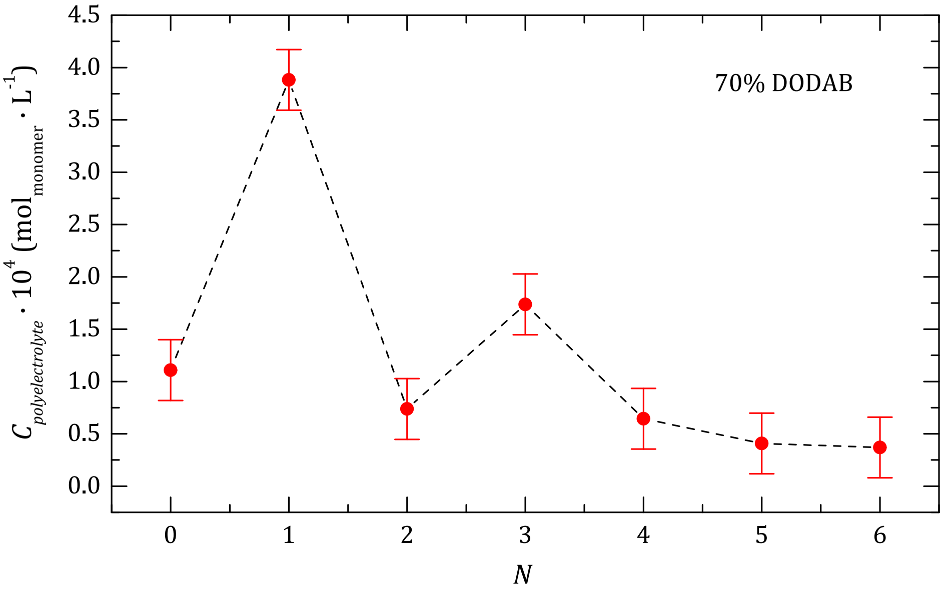

Figure S.1.- Amount of polyelectrolyte needed for reaching overcompensation as a function of the number of PSS/PAH layers deposited onto DOPC:DODAB liposomes with 30:70 compositional ratio.

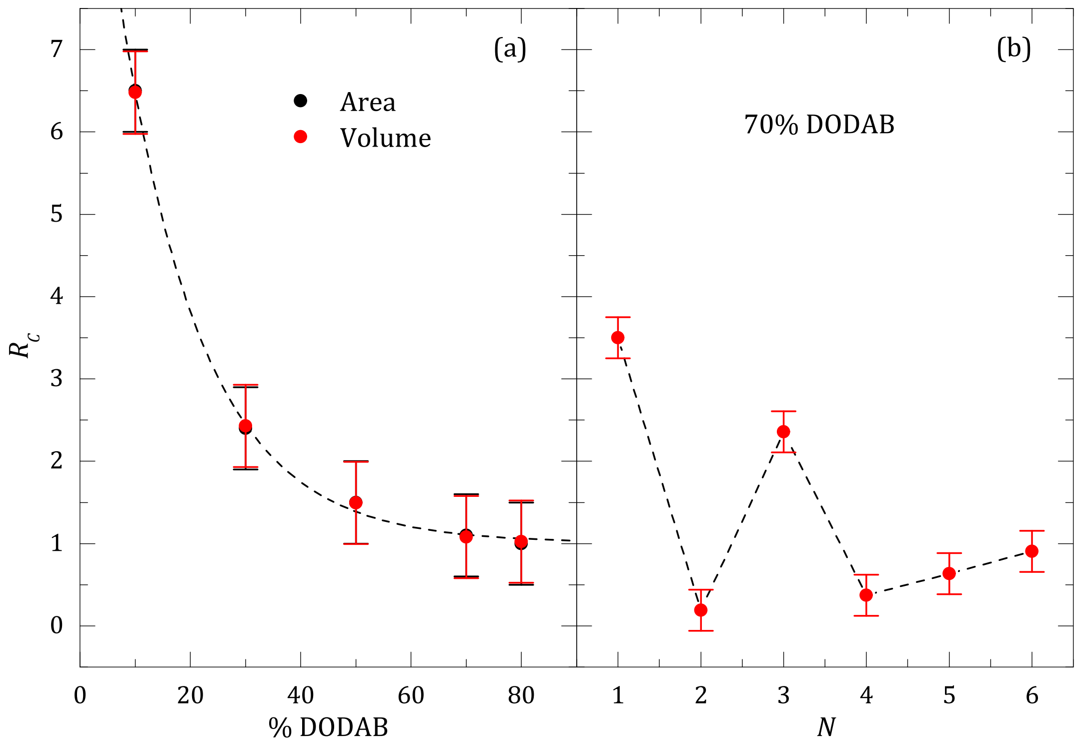

Figure S.2.- (a) Ratio between the number of deposited PSS monomers and charges on the external leaflet of the liposome, R_c, for full charge overcompensation as function of the weight fraction of DODAB in the

liposomes. The labels “area” and “volume” refer to the two ways to calculate the number of charges in the outer layer of the liposome, and the results coincide. (b) Effect of the number of layers on the overcompensation ratio, R_c, of polyelectrolyte monomers on each layer for the deposition of a PSS/PAH onto DOPC:DODAB liposomes with 30:70 compositional ratio.

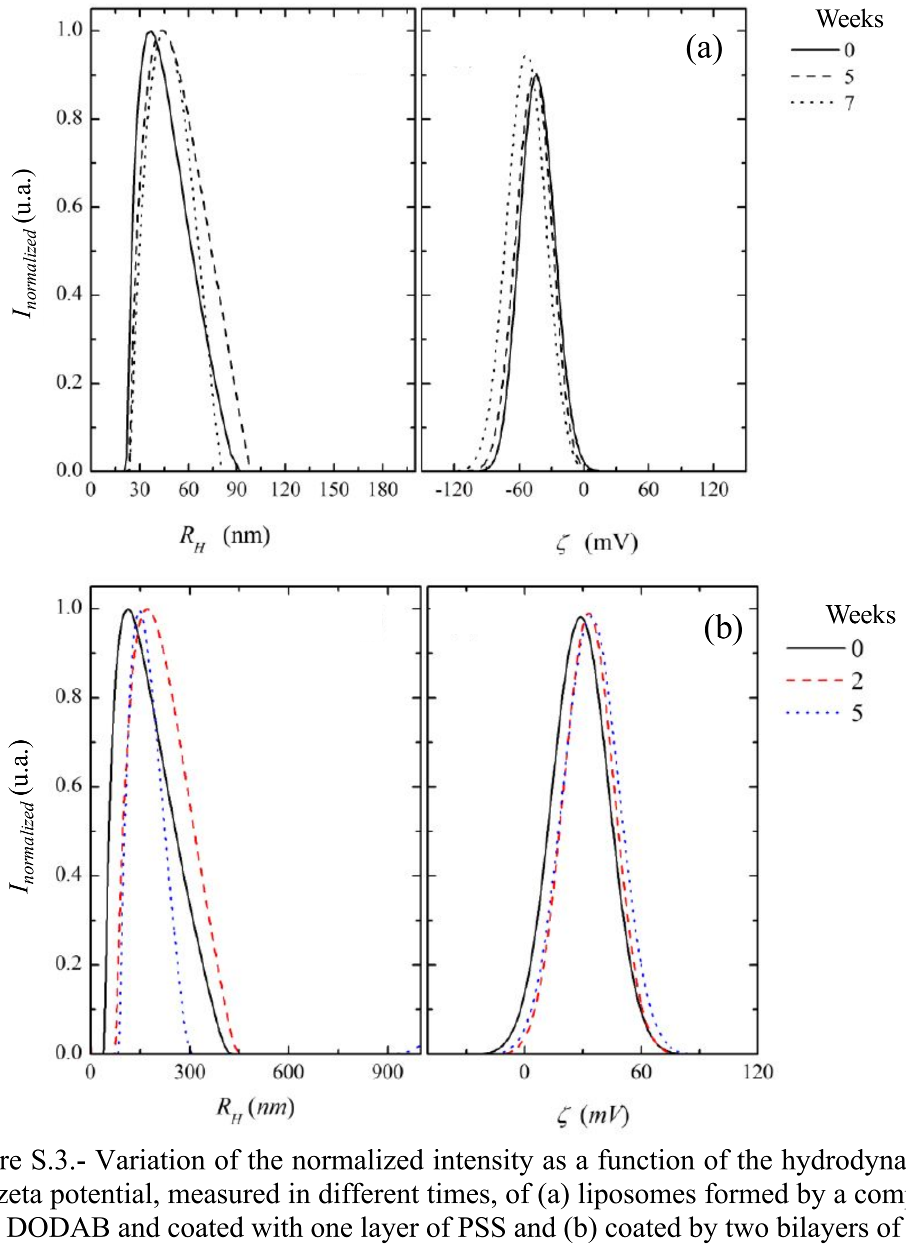

Figure S.3.- Variation of the normalized intensity as a function of the hydrodynamic radius and zeta potential, measured in different times, of (a) liposomes formed by a composition of 40% DODAB and coated with one layer of PSS and (b) coated by two bilayers of PSS/PAH.

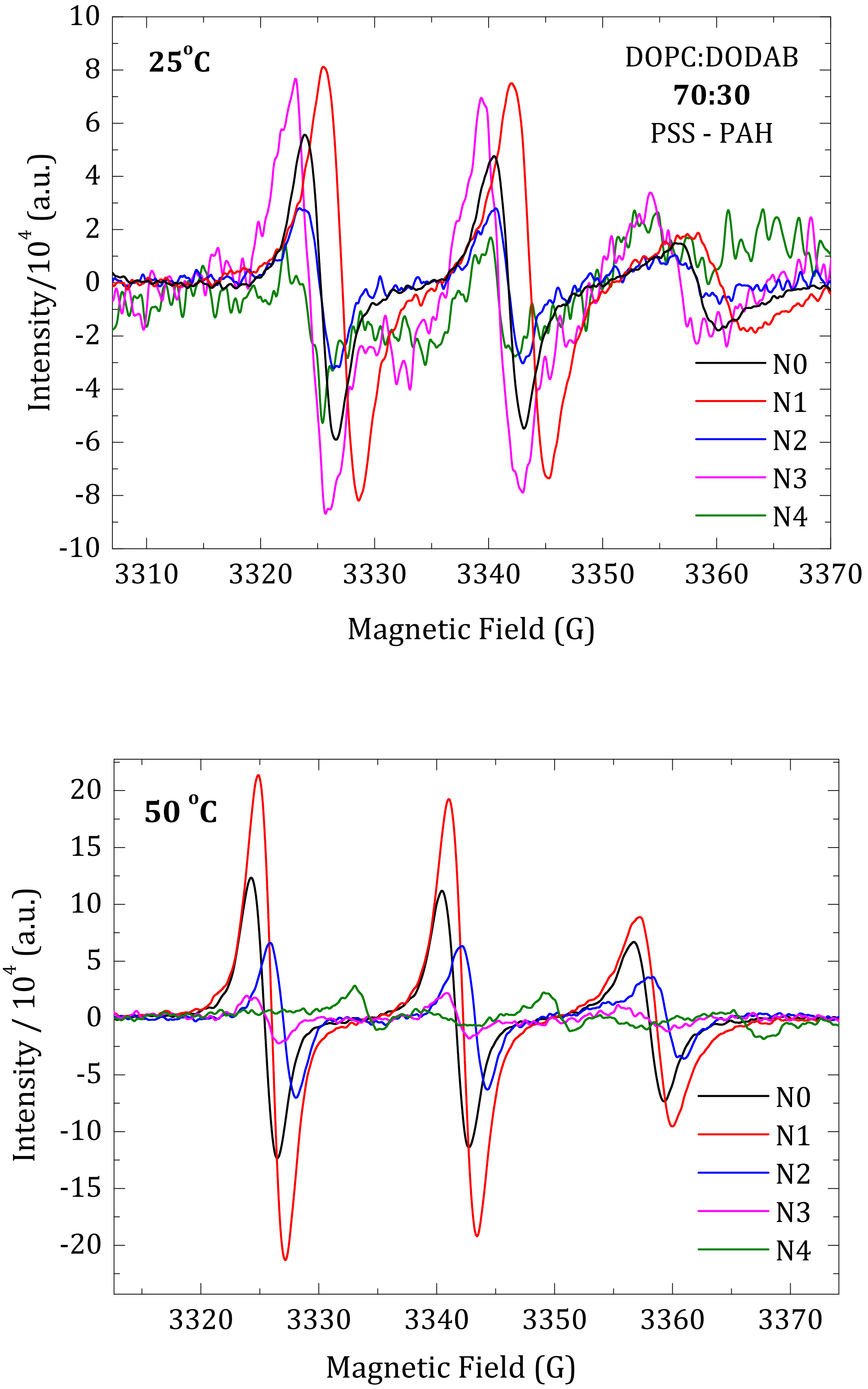

Figure S.4.- Set of ESR spectra for liposomes of DOPC:DODAB (70:30) coated with different number of layers as were obtained at two different temperatures (25º C and 50 ºC).
